# Survey of the specializing doctor training in orthopedics and traumatology across university hospitals in Finland

**DOI:** 10.1080/17453674.2021.1910772

**Published:** 2021-06-02

**Authors:** Sofianna Ojala, Heikki Kröger, Juhana Leppilahti, Juha Paloneva, Joonas Sirola

**Affiliations:** a Kuopio Musculoskeletal Research Unit (KMRU), University of Eastern Finland, and Department of Orthopedics, Traumatology and Hand Surgery, Kuopio University Hospital, Kuopio; b Department of Surgery, Division of Orthopedic and Trauma Surgery, Oulu University Hospital, Oulu, Finland/Medical Research Center, University of Oulu; c Central Finland Hospital, Jyväskylä, Finland, and University of Eastern Finland

This annotation describes the results from national audit of the orthopedics and traumatology specialization program and specializing physicians’ skills across all 5 university hospitals in Finland (Helsinki University Hospital, HUH; Kuopio University Hospital, KUH; Tampere University Hospital, TAYS; Turku University Hospital, TYKS; and Oulu University Hospital, OYS).

Competency-based training in surgical specialties is gathering more interest worldwide (Nousiainen et al. 2018, Gustafsson et al. 2019, LaPorte et al. 2019). In Finland, at the end of 2018, a reform of specializing physician training and also of the whole specialist training in surgery was launched, aiming at taking steps towards competency-based education (Paananen 2017, Seppänen 2018). The previous specialization curriculum was time dependent, taking 6 years of surgical training at minimum. It included 9 months in primary healthcare service, a minimum of 2 years and 3 months of general surgical training at a central hospital, after which more focused specialty training (such as orthopedics and traumatology) took place in a university hospital (3 years or more). The competency of a consultant orthopedic surgeon was then granted after finalizing the national specialization exam, consisting of 5 freely formulated questions concerning orthopedics and traumatology.

As a result of the reform in Finland, the narrower specialty of surgery must currently already be decided at the application phase. The actual specialization includes a 12-month surgical orientation period in various areas of surgery, followed by a 6-month trial period in a narrower specialty, such as orthopedics and traumatology. After the trial period, there is 9–12 months of competency-based general education in a narrower specialty and then a 3-year differentiating phase. At least 1 year of training must be completed at a university hospital and at least 1 year at a central hospital. Therefore, differences may be found in Finnish specialist training in comparison with other countries (reviewed earlier). For an example, in the United Kingdom (UK), Trauma & Orthopedic surgery training initially includes a 2-year Foundation Training in different specialties of medicine, and after that doctors apply for a Core Surgical Training (CST) program for the next 2 years. CST includes 4- to 6-month periods in different areas of surgery. After CST, junior surgeons apply for a Specialist Surgical Training program, which typically lasts 6 years and ends with a specialty exit exam. After passing the exam a Certificate of Completion of Training is received (BOTA Collaborations and Rashid 2018).

Compared with other European countries in addition to the UK, France and a few other countries do not have any mandatory course training, in comparison with Finland where 80 hours is required. On the other hand, in Croatia and Denmark the requirement is over 300 hours of course training. The highest minimum numbers of required surgical procedures are in the UK and Ireland—1800 procedures—whereas in Finland there are no specified requirements. In Finland there is a final written exam, but for example in Sweden there is no exam at all (Madanat et al. 2017). The present annotation provides extensive information on the different areas of specialization training in orthopedics and traumatology.

## Electronic survey

The electronic audit questionnaire (Supplement 1) was compiled for specializing physicians (registrars) in orthopedics and traumatology using the SurveyMonkey tool. The questionnaire was sent by e-mail link to all specializing physicians (n = 61) at the time of the audit, i.e., April to June 2019. All of these specializing physicians had completed the common trunk of their surgical training at the time of the audit. They are also part of the old system of specialist training before the reform at the end of 2018, when specialist training was time dependent, taking 6 years minimum, and general surgical training in various fields of surgery was 15 months in duration. Since the reform at the end of 2018, specialist training includes a 12-month orientation period in various fields of surgery and after that is focused only on the narrower specialty, for example orthopedics and traumatology, and is more competency-based than time dependent. The questionnaire included around 100 questions regarding surgical skills and education, clinical and scientific work, and other aspects of specializing physician training. The data was pseudonymized and the respondents gave permission to use the answers for research purposes.

The audit included 2 questions on the amount of and competency in orthopedic and traumatological procedures performed. These numbers were a subjective estimate made by the specializing physicians themselves. 9 respondents gave indefinite, non-numerical answers and were eliminated. 14 respondents gave answers such as “100–200” or “100+,” in which case we considered the mean of the range as the definite answer or the lowest reported number.

## Educational views (Supplement 2)

36 (mean age 35 years, 23 male) of 61 submitted surveys were answered. 3 respondents answered only the first question and were eliminated from the analyses.

22 respondents considered job description to be the most important factor when choosing a future job. Interestingly, all respondents intend to work as an orthopedist in a public hospital or facility in the future after the specialization program rather than the private sector.

26 respondents consider that university hospitals have a good or very good opportunities for accessing leadership training. However, 10 respondents consider the opportunities to be poor or very poor. According to the respondents, leadership training is offered for an average of 0–30 credits and is free of charge. Almost all (32) respondents have calendar time set aside for meeting-type training (approximately 3 hours per week). However, no working time is set aside for preparation of meeting presentations.

## Surgical skills training (Supplement 3)

When considering the traumatological procedures done by specializing physicians, all respondents have operated on a hip fracture with a trochanteric nail, operated on an ankle fracture, and 33 respondents have done a plate fixation of a wrist fracture independently in some way. In contrast, one-third have operated on a proximal humerus fracture and one-fifth have operated a vertebral fracture independently.

When considering the orthopedic procedures performed by specializing physicians independently, none of the respondents have operated on a knee cruciate ligament or collateral ligament with a graft and only 1 has done medial patellofemoral ligament reconstruction independently. One-fourth have done shoulder decompression independently and 5 respondents have operated on a rotator cuff rupture. In contrast, 34 respondents have removed osteosynthesis material independently and 33 have done carpal canal release independently.

## Synthesis of the survey

In this study, we audited the content of the specialist training program in Finland before the reform at the end of 2018. In this way, it will be possible to evaluate the success of renewed training in the future by implementing the survey again after 3–4 years. Most likely there will be changes in the duration of the specialization. Also, the number of independently performed surgical procedures may increase as the narrower specialty of surgery is already decided in the application phase and because the training is more competency oriented.

According to the present audit, all of the respondents intend to work as a specialist at a public hospital or facility in the future and none of the respondents are considering working in the private sector. In many countries, it is common to enter a fellowship after specialization in orthopedics and traumatology. This is not the case in Finland, and the interest in working in the public sector might be due to fact that the respondents want to gather more experience after graduation before working in the private sector. In Finland, specialization in orthopedics and traumatology does not officially include a working period in the private sector. Accordingly, this may influence reluctance to consider a private hospital as a future employer.

Specializing physicians gave a self-estimated number of how many independently performed procedures they have done already. A common logbook at the national level is paramount to obtain more exact information on the true number of procedures. At present, steps at the national level have been taken to introduce such a uniform logbook.

Recent evidence favors a nonoperative treatment line for several orthopedic conditions. As an example, the number of independently performed surgeries on proximal humerus fractures was quite low, which may reflect treatment policies. Also, the number of arthroscopic procedures was low, reflecting recent evidence.

The overall response rate was modest. Two-thirds of the specializing physicians in Finland responded to the survey, but this sample can be considered quite representative as all university hospitals were included.

This audit did not include a section on pediatric orthopedics. Pediatric orthopedics is a subspecialty in Finland and is not provided in all university hospitals due to lack of resources. The purpose in this annotation was to audit basic training in orthopedics and traumatology provided at all university hospitals.

In conclusion, according to our survey of the orthopedic specialization in Finland, the number of key orthopedic procedures was found to be quite high. The survey also provides widespread information on the general training conditions of specializing physicians in orthopedics and traumatology in Finland. In the future, auditing will be easy to extend to other areas of medical specialization too. The information can be used directly to develop the structure and content of specialist training. In the first instance, the procedures should be taught according to evidence-based medicine. According to the results of the questionnaire, the amount of arthroscopy training should be increased. Also, new audits in other countries can be compared to further develop specializing-doctor training. The effect of the renewal on specialization training remains to be seen after follow-up audits. Arthroscopy training may be improved by modern VR (virtual reality) based simulators. Also, other VR surgical training is evolving and may substantially change the training in widespread areas of orthopedics and traumatology.

## Supplementary data

Supplements 1–3 are available in the online version of this article, http://dx.doi.org/10.1080/17453674.2021.1910772
